# The Relationship Between Bone Health Status of Post-Menopausal Women with Non-Functional Adrenal Tumours/Mild Autonomous Cortisol Secretion and Their Baseline Morning Adrenocorticotropic Level

**DOI:** 10.3390/diagnostics16020180

**Published:** 2026-01-06

**Authors:** Alexandra-Ioana Trandafir, Oana-Claudia Sima, Nina Ionovici, Dana Manda, Mihai Costachescu, Mara Carsote

**Affiliations:** 1 PhD Doctoral School of “Carol Davila” University of Medicine and Pharmacy, 020021 Bucharest, Romania; alexandra-ioana.trandafir@drd.umfcd.ro (A.-I.T.); carsote_m@hotmail.com (M.C.); 2 Department of Clinical Endocrinology V, “C.I. Parhon” National Institute of Endocrinology, 011863 Bucharest, Romania; oana-claudia.sima@drd.umfcd.ro; 3 Occupational Medicine Department, University of Medicine and Pharmacy of Craiova, 200349 Craiova, Romania; 4 Department of Research, “C.I. Parhon” National Institute of Endocrinology, 011863 Bucharest, Romania; dana.manda@parhon.ro; 5 Department of Radiology and Medical Imaging, “Dr. Carol Davila” Central Military University Emergency Hospital, 010825 Bucharest, Romania; mihai.costachescu@drd.umfcd.ro; 6 Department of Endocrinology, “Carol Davila” University of Medicine and Pharmacy, 020021 Bucharest, Romania

**Keywords:** DXA, bone turnover marker, FRAX, diabetes, mild autonomous cortisol secretion, dexamethasone test, FRAXplus, cortisol, hormone, endocrine

## Abstract

**Background**. Glucocorticoid-induced osteoporosis represents a well-known type of secondary osteoporosis (SOp). While the most prevalent sub-category includes corticotherapy, another important contributor is represented by Cushing’s syndrome. In this traditional landscape, adrenal incidentalomas do not involve a standard cause of SOp, since most of them are non-functioning adrenal tumours (NFATs). Yet, 30–40% of them are not entirely “non-functioning”, due to mild autonomous cortisol secretion (MACS). Despite not being a guideline-based diagnosis, a lower ACTH might point to various NFATs/MACS complications. **Objective**. This study aimed to determine the relationship between the bone health status of post-menopausal women with NFATs/MACS and their baseline morning ACTH level. The bone health indicators were DXA, FRAX, and bone remodelling markers. **Methods**. This was a retrospective, real-life, transversal study in adult females who were hospitalized in a single tertiary centre of endocrinology. They were all anti-osteoporotic drug-naïve. The subjects underwent CT and DXA scanning and a 1 mg dexamethasone suppression test (DST). **Results**. The cohort (sample size of N = 84 patients, 61.49 ± 7.86 years) had a type 2 diabetes rate of 18%, arterial hypertension rate of 75%, and a dyslipidemia rate of 78%. Median ACTH was 11.89 pg/mL. The prevalence of MACS was 30.95%. The mean largest tumour diameter (LTD) was 2.25 ± 0.99 cm. ACTH correlated with second-day cortisol after the 1 mg DST (r = −0.301, *p* = 0.024), and LTD (r = −0.434, *p* < 0.001). ROC analysis for the bone resorption marker CrossLaps showed an AUC of 0.647 (*p* = 0.05), with the highest Youden index for the cut-off at 0.32 ng/mL (sensitivity 87.50%, specificity 39.50%). Bone impairment (osteoporosis + osteopenia) was found in 65% of patients, with an osteoporotic fracture prevalence of 4.76%. The lowest mean T-score (−1.12 ± 1.00) showed osteopenia, and the median trabecular bone score pointed a partially degraded microarchitecture [median (interquartile interval): 1.320 (1.230, 1.392)]. FRAX and FRAXplus estimations correlated with bone mineral density (BMD) at all three central DXA sites, regardless of the ACTH cut-off. Patients with a low ACTH (<10 pg/mL) displayed similar bone/adrenal features when compared to those with normal ACTH, except forbut they had a higher MACS rate (45.45% versus 21.57%, *p* = 0.021) and a larger LTD (2.67 ± 0.98 versus 1.98 ± 0.92 cm, *p* = 0.003). Fracture estimation showed that only in patients with a low ACTH, the 10-year fracture risk for major osteoporotic fractures (MOF) adjusted for lumbar BMD was lower than the risk for MOF adjusted for diabetes (*p* = 0.036), and the 10-year hip fracture risk was lower when adjusted for lumbar BMD (*p* = 0.007). ACTH correlated with lumbar BMD (r = 0.591, *p* = 0.002) only in the group with an ACTH < 10 pg/mL, suggesting its potential usefulness as a bone biomarker in these cases. On the other hand, MACS-negative subjects with a low ACTH versus those with a normal ACTH showed higher CrossLaps (0.60 ± 0.27 versus 0.42 ± 0.21 ng/mL, *p* = 0.022), indicating an elevated bone resorption even in patients with tumours that are regarded as true non-secretors. **Conclusions**. A subgroup of patients diagnosed with NFATs/MACS might be prone to skeletal damage, and biomarkers such as ACTH (specifically, suppressed ACTH) might serve as a surrogate pointer to help refine this higher risk in daily practice. Further research to address other ACTH cut-offs will place ACTH assays in the overall bone status evaluation in these patients, most probably not as a single biomarker, but in addition to other assays.

## 1. Introduction

Glucocorticoid-induced osteoporosis represents a well-known type of secondary cause of bone loss [1,2]. While the most prevalent sub-category includes corticotherapy, another important (endocrine) contributor is represented by Cushing’s syndrome caused by cortisol-producing adrenal tumours, by adrenocorticotropic hormone (ACTH)–secreting pituitary tumours (Cushing’s disease), or, to a lesser extent, by paraneoplastic Cushing’s syndrome accompanying various malignancies (e.g., lung out cell carcinoma, medullary thyroid cancer, or other neuroendocrine neoplasia) [3,4,5,6,7,8,9,10].

**Figure 1:**
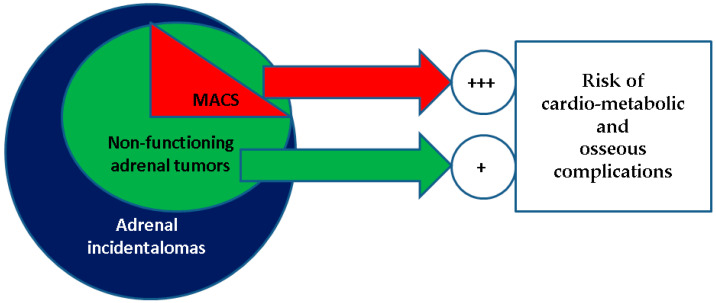
Visual representation of a MACS-positive profile in the general category of non-functioning adrenal tumours, which represents the most common type of adrenal incidentaloma; both MACS-positive and the entire group of non-functioning adenomas are associated with an increased risk of cardio-metabolic and osseous (osteoporosis, osteopenia, fragility fractures) complications when compared to with healthy controls, with a higher rate for MACS-positive versus MACS-negative adenomas.

In this rather traditional landscape of cortisol-related bone impairment, adrenal incidentalomas (in apparently healthy individuals in whom an adrenal tumour is accidentally detected through imaging procedures for non-adrenal ailments) do not involve a standard cause of secondary osteoporosis [11,12,13]. Most of them are generally designated as “non-functioning adrenal tumours” (NFAT). One-third is not entirely “non-functioning”, since mild autonomous cortisol secretion (MACS) has been reported [14,15,16] (Figure 1).

Both MACS-positive and MACS-negative adrenal incidentalomas are associated with increased risks of various osseous and non-osseous ailments, noting that an adrenal incidentaloma is found in 1% to 5% of all imaging procedures [17,18,19,20,21]. For instance, Favero et al. [22], in a meta-analysis from 2024, showed that patients with NFATs versus controls (non-NFATs) had a statistically significant correlation with the presence of arterial hypertension (odds ratio of 1.87, 95% confidence interval between 1.39 and 2.51), composite diabetes mellitus index (odds ratio of 2.04, 95% confidence interval between 1.7 and 2.45), and metabolic syndrome (odds ratio of 2.89, 95% confidence interval between 1.93 and 4.32) [22]. According to another meta-analysis that was published in 2023, Pelsma et al. [23] analyzed 30 cross-sectional and 16 cohorts (enrolling a total of 17,156 subjects) and identified that individuals with MACS-positive tumours had a statistically significant relative risk of diabetes of 1.44, hypertension of 1.24, and dyslipidaemia of 1.23. After adjustment for confounders, MACS was associated with a higher all-cause mortality, as reflected by a hazard ratio of 1.54 [23]. Notably, osteoporosis (and abnormal DXA results) was reported in 20–40% of patients with NFATs and in 40–60% of patients in certain MACS-positive subgroups [24].

A current gap in daily practice is represented by the panel of confirmatory biomarkers of mild endocrine tumour activity, which at present, from a guideline-based perspective, is based on a 1 mg overnight screening suppression dexamethasone test (DST) [25,26,27]. A non-functional profile is considered at a second-day post-DST cortisol value of less than 1.8 µg/dL, while MACS is positive at a blood cortisol level between 1.8 and 5 µg/dL (in the absence of the overt Cushing’s syndrome–related clinical features) [28,29,30]. Over the years, the terms “subclinical Cushing’s syndrome” and “subclinical hypertcortisolism” were no longer recommended, while the criteria of diagnosis also varied, which might explain the heterogeneous spectrum of the studies’ results over the years [31,32,33,34,35,36,37]. With respect to bone status and glucocorticoid exposure, the use of DXA in terms of bone mineral density and trabecular bone score has been proved useful [38,39,40].

Despite not being a guideline-based diagnosis, lower ACTH have been found as feedback to the small cortisol excess from the adrenal tumour/incidentalomas. Yet, not all NFATs express this profile (but a higher prevalence was observed in MACS-positive when compared with MACS-negative adenomas, according to many studies, but this has not been unanimously confirmed) [41,42,43]. ACTH assay is currently regarded as an additional clue of mild endocrine secretion, as, similarly, it has been proposed for the suppressed value of adrenal androgens [44,45,46]. As mentioned, even a MACS-negative adenoma might present a decreased baseline morning ACTH, which suggests some limits of the current MACS characterization amid DST testing [47,48,49,50].

Specifically, a low ACTH includes a completely suppressed (even undetectable) blood level, a value below the lower upper normal, or a low-normal assay below certain cut-offs (e.g., 10-to-15 pg/mL), which are not standardized yet, and do not represent a guideline-based perspective. This assessment belongs to a patient-centred approach and may become a fine index for the identification of cortisol-related comorbidities, as well as a pointer for further management (e.g., adrenalectomy or conservative approach) in patients diagnosed with NFAT/MACS [51,52,53]. Notably, the importance of adrenal incidentalomas starts from a more frequent diagnosis in relation to more frequent imaging procedures during the recent COVID-19 pandemic and goes into a deep perspective of multimodal complications and tumour categories, as found in NFATs/MACS [54,55,56,57,58,59,60,61,62,63,64].

### Objective

This study aimed to determine the relationship between the bone health status of post-menopausal women with NFATs/MACS and their baseline morning ACTH level. The bone health indicators were DXA, FRAX, and bone remodelling markers. We analyzed the rate of suboptimal bone health status, the relationship between bone health status and adrenal biomarkers (hormonal and imaging panel—ACTH, cortisol, largest tumour diameter), and the differences in bone health between subjects with different ACTH levels, specifically at the 10 pg/mL cut-off.

## 2. Material and Methods

### 2.1. Study Design

This was a retrospective, real-life, transversal study in adult females diagnosed with adrenal incidentalomas who were hospitalized in a single tertiary centre of endocrinology.

### 2.2. Study Population

The subjects were analyzed according to the following inclusion and exclusion criteria (Table 1).

### 2.3. Study Protocol

**Figure 2:**
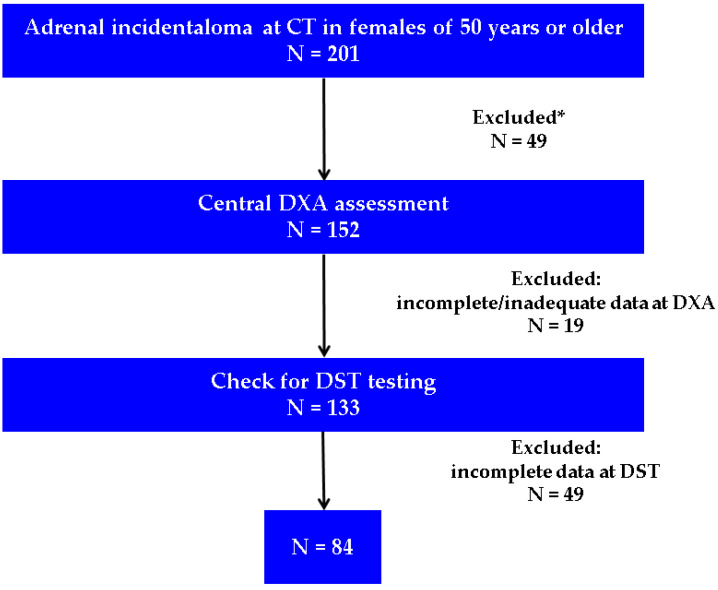
Flowchart of the study protocol (Abbreviations: CT = computed tomography; DXA = Dual-Energy X-Ray Absorptiometry; DST = dexamethasone suppression test; N = number of patients; * involves the exclusion criteria from Table 1; incomplete data at DST were considered if the timing of blood registration at the hospital lab system was other than 6:00 to 7:30 a.m.).

Menopausal women over 50 years in whom an adrenal incidentaloma was discovered during computed tomography (CT) were assessed for exclusion criteria and checked for available DXA and DST results (Figure 2).

The assessments included demographic features, body mass index (BMI) calculation (kg/sqm), blood assays, CT-based features (unilateral/bilateral adrenal tumour/tumours and the largest tumour diameter), DXA parameters, and FRAX (Fracture Risk Assessment Tool)–based osteoporotic fractures probabilities (Table 2).

Medical records and current hospitalization provided age (years); years since menopause; prior/current diagnosis of type 2 diabetes mellitus, arterial hypertension, and dyslipidaemia; and prior medical history, including prevalent fragility fractures. The mineral metabolism assays included total serum calcium, phosphorus, magnesium, parathyroid hormone (PTH), 25-hydroxyvitamin D (25OHD), and blood bone turnover markers [total alkaline phosphatase, osteocalcin, P1NP (procollagen type 1 N-terminal pro-peptide), and CrossLaps].

Central DXA provided a bone mineral density (BMD)/T-score and, thus, traditional World Health Organization (WHO) categories of osteoporosis (lowest central T-score of less than or equal to −2.5) and osteopenia (T-score between −1 and −2.5) were identified [65]. We designated the group “bone impairment” for the patients with non-normal BMD (osteoporosis + osteopenia). A lumbar DXA scan provided the trabecular bone score (TBS). FRAX [66] allowed the calculation of 10-year major osteoporotic fracture risk (MOF)/hip fracture risk (HF) with femoral neck BMD, and FRAXplus [67] allowed MOF/HF adjustment for lumbar BMD and type 2 diabetes, respectively. Prevalent (osteoporotic, low-trauma, or spontaneous) fragility fractures were assessed based on medical records and a screening X-ray of the thoracic and lumbar spine (for vertebral fractures). CT, DXA, and X-Ray scans were re-analyzed by a trained radiologist who provided the second opinion of the mentioned study parameters (M.K.)

DST included baseline plasma (fasting) cortisol and ACTH, as well as the second-day cortisol after 1 mg dexamethasone administration. NFAT was designated for all cases with second-day cortisol < 5 µg/dL, and among NFATs, the MACS-positive profile was designated for those with a second-day cortisol > 1.8 and <5 µg/dL. We applied another designation for the group with “suppressed ACTH”, which included individuals with a blood baseline ACTH lower than 10 pg/mL (the threshold was based on prior published data) [68,69,70,71,72], and a comparative analysis between group S and nonS (with ACTH ≥ 10 pg/mL) was performed.

### 2.4. Statistical Analysis

SPSS v.29.0.2.0 (IBM, Armonk, NY, USA), Excel v.16.101 (Microsoft, Redmond, WA, USA), and GraphPad Prism v.10.6.0 (GraphPad Software, Boston, MA, USA) were used to perform the statistical analysis. Continuous variables were reported as mean ± standard deviation (SD) for normal distributions or median (M) and quartiles (Q1, Q3) for non-normal distributions. Categorical variables were presented as counts and percentages. The distribution type of numerical variables was assessed using the Kolmogorov–Smirnov test and visual inspection of histograms. Correlation between numerical variables was evaluated using the Spearman coefficient. Differences between categorical variables were assessed using the Chi-square test or Fisher’s exact test. Comparisons of continuous variables between two independent groups were performed using the independent samples *t*-test when assumptions of normality and homogeneity of variance were met, and the non-parametric Mann–Whitney U test was applied otherwise. Receiver operating characteristic (ROC) curves were generated to evaluate the association between numerical variables and outcomes, and the area under the curve (AUC) was calculated to assess discriminatory performance. The optimal cut-off value was identified using the Youden index. Multiple linear regression was used to identify independent predictors, and results were reported as unstandardized coefficients ± standard error (B ± SE) and standardized coefficients (β). The selection of independent variables for the multiple linear regression model was based on the unadjusted results of the univariate analyses performed on the dataset. The coefficient of determination (R-squared) was used to assess the explanatory power of the model. A two-tailed *p*-value < 0.05 was considered statistically significant for all tests.

### 2.5. Ethical Approval

The Ethical Committees approved the retrospective data collection as follows: Carol Davila University of Medicine and Pharmacy, Bucharest, Romania (7634-04/04/2025) and “C.I. Parhon” National Institute of Endocrinology, Bucharest, Romania (37-09/22/2025).

## 3. Results

### 3.1. NFATs’ Characterization

A total of 84 patients with a mean age of 61.49 ± 7.86 years were evaluated. Type 2 diabetes mellitus was diagnosed in 18.07% of the women, arterial hypertension in 75.00%, and dyslipidemia in 78.57%. BMI had a mean value of 29.48 ± 5.70 kg/sqm. The endocrine panel showed a median ACTH of 11.89 pg/mL; suppressed ACTH (baseline value < 10 pg/mL) was found in 39.39% of the entire group, MACS was found in 30.95% of the entire cohort, and the largest tumour diameter had a mean value of 2.25 ± 0.99 cm (Table 3).

In the cohort, 4.76% of patients had prevalent fragility fractures and 65.48% had bone impairment (osteoporosis + osteopenia) (Table 4).

Statistically significant negative correlations were found between baseline ACTH with second-day plasma cortisol after 1-mg DST (r = −0.301, *p* = 0.024), respectively, with largest tumour diameter (r = −0.434, *p* < 0.001). Post-DST cortisol statistically, significantly, and positively correlated with the largest tumour diameter (r = 0.572, *p* < 0.001) (Table 5).

**Figure 3:**
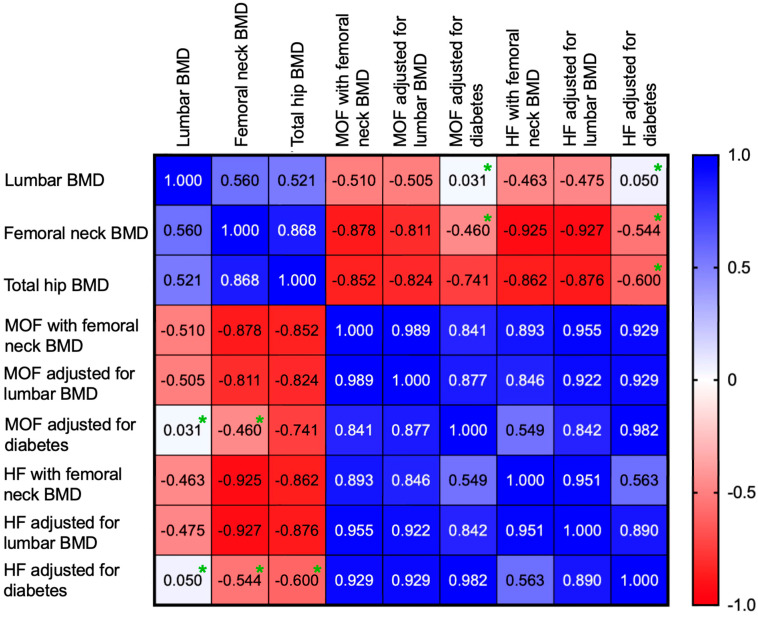
Heatmap showing the correlation coefficients between lumbar BMD, femoral neck BMD, total hip BMD and 10-year fracture risk score (green asterisk = correlations with *p* > 0.05).

MOF, HF with femoral neck BMD and MOF, HF adjusted for lumbar BMD were negatively correlated with lumbar BMD, and total hip BMD. (Table 6, Figure 3).

### 3.2. Analysis Depending on ACTH Threshold

The entire cohort was assessed based on two groups—group S (N = 33) and nonS (N = 51)—depending on the ACTH cut-off of 10 pg/mL (<10 versus ≥10 pg/mL). Age sub-analysis showed a similar rate of patients in each group (*p* = 0.146) (Table 7).

**Figure 4:**
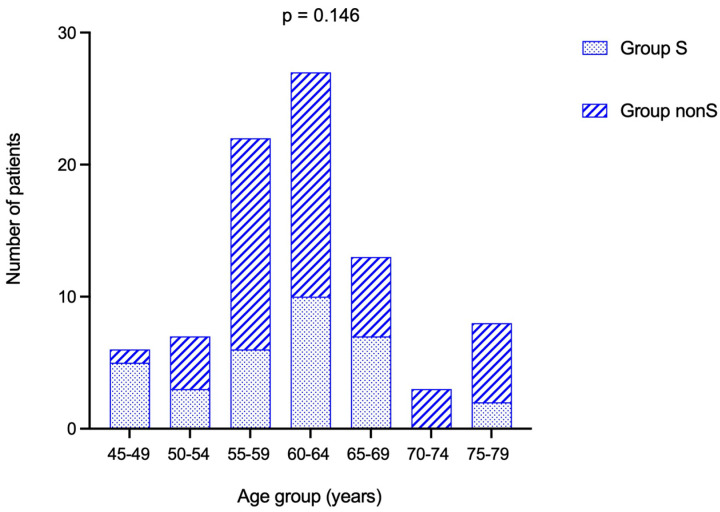
Stacked bar chart showing number of patients from group S and group nonS in each age-group.

Most patients were found in the sub-category between 60 and 64 years (Figure 4).

Demographic parameters were similar between group S and group nonS. The prevalence of MACS was statistically significantly higher in group S versus nonS (*p* = 0.021). The largest tumour diameter was statistically significantly elevated in group S (2.67 ± 0.98 cm) versus group nonS (of 1.98 ± 0.92 cm, *p* = 0.003) (Table 8).

Second-day plasma cortisol after 1 mg DST was statistically significantly higher in group S versus nonS [2.08 (1.58, 3.08) versus 1.25 (0.94, 2.02) µg/dL, *p* = 0.014].

An ROC curve plotted sensitivity and 1-specificity of the largest tumour diameter for predicting suppressed ACTH (<10 pg/mL), with a statistically significant AUC of 0.702 (*p* = 0.003). The Youden index was computed for each cut-off value, and the highest index yielded a sensitivity of 63.30% with a 95% confidence interval (CI) between 45.51 and 78.13 and a specificity of 75.00% (95% CI: 61.22–85.08), corresponding to a cut-off value of 2.45 cm (Table 9).

**Figure 5:**
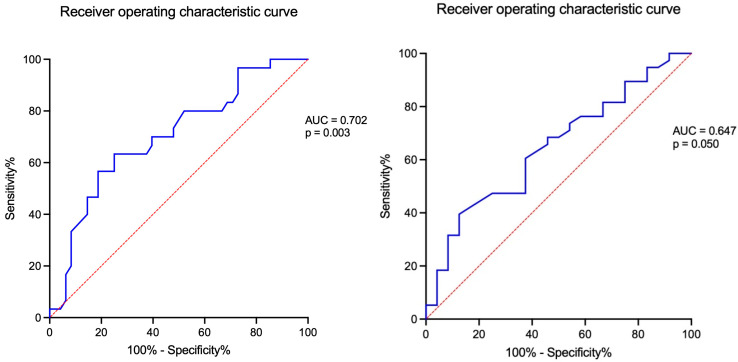
ROC curve of the largest tumour diameter (left) and the CrossLaps level (right) for predicting a suppressed blood ACTH (<10 pg/mL).

Using ROC curve analysis, sensitivity and 1-specificity values of CrossLaps were examined for the ability to predict suppressed ACTH, with a statistically significant AUC of 0.647 (*p* = 0.050). The highest Youden index corresponded to a cut-off value of 0.32 ng/mL, with a sensitivity of 87.50% (95% CI: 69.00–95.66) and specificity of 39.50% (95% CI: 25.60–55.28) (Figure 5).

The rate of prevalent fragility fractures and DXA-based bone impairment, as well as 10-year fracture risk estimations were similar between group S and group nonS (Table 10).

Bone formation marker alkaline phosphatase had a statistically significantly higher value in group S versus nonS (91.15 ± 38.84 U/L versus 73.49 ± 24.30 U/L, *p* = 0.017), as did bone resorption marker CrossLaps (0.56 ± 0.29 ng/mL versus 0.44 ± 0.22 ng/mL, *p* = 0.05).

MOF (calculated with femoral neck BMD) was statistically significantly higher when compared to MOF adjusted for lumbar BMD within each of the groups (*p* < 0.001 for each). MOF adjusted for diabetes was statistically significantly higher than MOF adjusted for lumbar BMD only within group S (*p* = 0.036) (Table 11).

HF calculated with femoral neck BMD was statistically significantly higher than HF adjusted for lumbar BMD only in group S (*p* = 0.007).

Group S showed a statistically significant positive correlation between second-day plasma cortisol after 1 mg DST and the largest tumour diameter (r = 0.591, *p* = 0.002) and between baseline ACTH and lumbar BMD (r = 0.434, *p* = 0.024). On the other hand, group nonS was found to have statistically significant correlations between baseline ACTH and morning plasma (baseline) cortisol (r = 0.315, *p* = 0.028), between ACTH and the largest tumour diameter (r = −0.395, *p* = 0.005), and between baseline cortisol and total hip BMD (r = −0.391, *p* = 0.033) (Table 12).

Both group S and group nonS confirmed that MOF/HF calculated with femoral neck BMD (FRAX) and MOF/HF adjusted for lumbar BMD (FRAXplus) were statistically significantly and negatively correlated with lumbar BMD, femoral neck BMD, and total hip BMD (Table 13).

The multivariate linear regression model for baseline ACTH had an R-squared of 0.225 (*p* = 0.016). The intercept corresponded to an ACTH value of 13.65 ± 7.32 pg/mL, which was not statistically significant (*p* = 0.079). The largest tumour diameter was identified as a statistically significant negative predictor of baseline ACTH, with each 1 cm increase in size associated with a 3.07 ± 0.98 pg/mL decrease in baseline ACTH (*p* = 0.003). Morning plasma cortisol, MACS-positive profile, age, and BMI were not statistically significant predictors of baseline ACTH (Table 14).

### 3.3. Analysis Depending on ACTH Threshold in Addition to MACS-Positive Profile (Based on Second-Day Cortisol After 1 mg DST)

**Figure 6:**
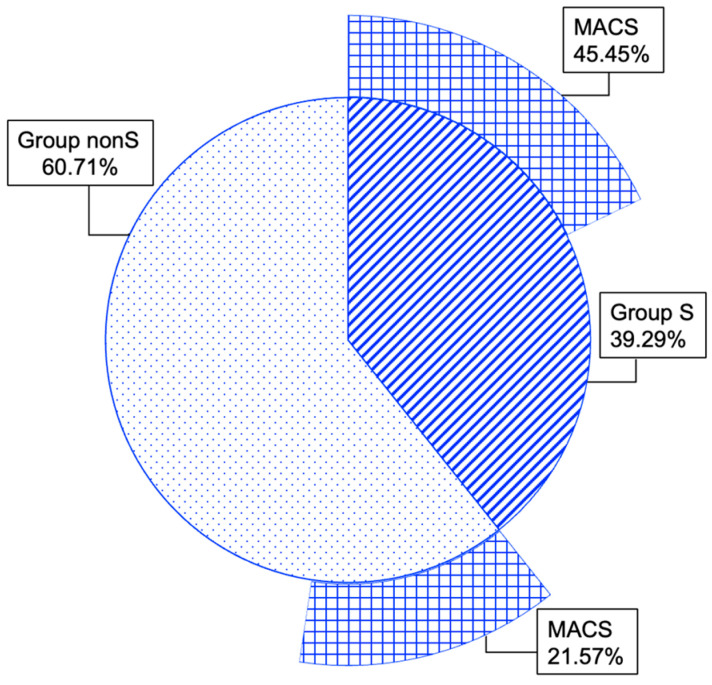
Multi-level doughnut chart showing percent of patients with MACS-positive features within group S and group nonS.

As mentioned, the entire cohort was split into group S (39.29%) and group nonS (60.71%). Additionally, MACS-positive tumours within groups S and nonS showed a rate of 45.45% and 21.57%, respectively, with a statistically significant difference (*p* = 0.021) (Figure 6).

Within group S, the prevalence of diabetes was statistically significantly higher in the MACS versus the nonMACS subgroup (40.00% versus 5.56%, respectively; *p* = 0.03), and an increase in BMI (*p* = 0.011) was observed. Within both groups, the largest tumour diameter was statistically significantly increased in MACS versus non-MACS (*p* < 0.008 and *p* = 0.039, respectively). The sub-analysis between MACS + low ACTH versus MACS+ normal ACTH showed similar results (Table 15).

Bone status evaluation (including the estimation of the fracture risk) was similar in the MACS-focused sub-analysis, except at the CrossLaps level, which was statistically significantly higher in individuals with nonMACS + low ACTH versus nonMACS + normal ACTH (Table 16).

## 4. Discussion

### 4.1. Study-Focused Analysis

We analyzed a cohort of menopausal females with NFATs/MCAS (N = 84, age of 61.49 ± 7.86 years). The endocrine panel showed an ACTH of 13.51 ± 8.29 pg/mL, while 39.39% of the subjects had an ACTH < 10 pg/mL, and MACS confirmation was detected in one-third of the entire cohort (a rate that is similar to that in prior published data [18,19]). Of note, at this point, an ACTH < 10 pg/mL is not standardized and is highly assay-dependent. A suppressed ACTH may reflect subtle autonomous cortisol secretion but cannot differentiate MACS from false positives.

Bone impairment based on a DXA assessment (non-normal BMD) was found in 65% of the cases, with a fragility fracture prevalence of 4.76%. The values of bone turnover markers showed a homogenous distribution, with means within normal blood ranges. Median TBS pointed to a partially degraded microarchitecture (1.320). The lowest mean T-score was at the femoral neck, highlighting osteopenia ranges (−1.12 ± 1.00). Notably, women who were already under specific anti-osteoporotic medication were ruled out, and, hence, the patients with osteoporosis have been underestimated in this cohort. The study population showed the expected associations between FRAX/FRAXplus-based estimations on the one hand and DXA-BMDs on the other hand, as found in the general population [65,73]. The highest median of 10-year fracture probabilities upon FRAX/FRAXplus models was for MOF calculated with a femoral neck BMD (3.9%).

ACTH inversely correlated with the largest tumour diameter (r = −0.434, *p* < 0.001), as well as the second-day plasma cortisol after 1 mg DST (r = −0.301, *p* = 0.024), but not with BMDs, TBS, or FRAX-based estimations. We conducted an analysis in groups based on the 10 pg/mL threshold for baseline ACTH. Previous research had reported that a cut-off of 10 pg/mL (2.2 pmol/L) associated high specificity (92.6%) with a sensitivity of 55.6% [68,69,70,71,72]. ACTH levels below this threshold have been suggested as additional diagnostic biomarker criteria for MACS, which is still an open matter [69,70,71]. An ROC curve plotted sensitivity and 1-specificity of the largest tumour diameter for predicting this suppressed ACTH level (<10 pg/mL) [AUC of 0.702 (*p* = 0.003)]. The Youden index was the highest for 2.45 cm (sensitivity 63.30%, specificity 75.00%). Moreover, ROC analysis for the bone resorption marker CrossLaps showed an AUC of 0.647 (*p* = 0.05), with the highest Youden index for the cut-off 0.32 ng/mL (sensitivity of 87.50%, specificity of 39.50%). Multivariate regression showed that only the largest tumour diameter was a statistically significant predictor of baseline ACTH, with each 1 cm increase in size associated with a 3.07 ± 0.98 pg/mL decrease in baseline ACTH (*p* = 0.003).

Group S versus nonS showed a similar age distribution, as well as did the panel of studied comorbidities and biochemical and hormonal assays, except for a higher MACS rate (45.45% versus 21.57%, *p* = 0.021), an increased largest tumour diameter (2.67 ± 0.98 versus 1.98 ± 0.92 cm, *p* = 0.003), and elevated second-day cortisol after DST [2.08 (1.58, 3.08) versus 1.25 (0.94, 2.02) µg/dL, *p* = 0.014]. Despite these mentioned endocrine anomalies (which imply adrenal status may be a potential source of the bone damage), the mineral metabolism exploration, DXA-BMD, TBS, and FRAX-based estimations were similar, noting that total alkaline phosphatase was elevated in group S versus nonS, with intra-normal average values (91.15 ± 38.84 U/L versus 73.49 ± 24.30 U/L, *p* = 0.017). Of note, BMD-FRAX correlations remained statistically significant, as previous studies described them in non-NFAT individuals [72,73,74].

Interestingly, MOF was statistically significantly lower when an adjustment for lumbar BMD was performed in both groups (*p* < 0.001), while MOF adjusted for lumbar BMD was lower than MOF adjusted for diabetes in group S (*p* = 0.036). HF was lower when adjusted for lumbar BMD within group S, as well (*p* = 0.007). These results, hence, suggest that FRAXplus might be a more useful tool in these patients (than the traditional FRAX algorithm, which provided MOF and HF), since differences between FRAX and FRAXplus estimations might be expected. Moreover, these differences seem more important in the group with lower ACTH, and a refinement of the 10-year fracture risk probability should take into consideration an ACTH threshold, but further validation by analyzing incidental fractures across longitudinal observations is mandatory in this specific matter, and only limited data have been published so far.

Another discordance in the interpretation of adrenal-bone assessment is reflected by the fact that ACTH is statistically significantly correlated with lumbar BMD in the group with suppressed ACTH, but not in the one with normal ACTH (r = 0.591, *p* = 0.002); thus, it might serve as a biomarker of bone impairment in menopausal women with NFATs/MACS, if baseline value is less than 10 pg/mL. Other possible explanations include differential cortisol sensitivity, the influence of the tumour size, or distinct adrenal venous drainage patterns.

Within group S, the prevalence of diabetes was statistically significantly higher in the MACS versus the nonMACS subgroup (40.00% versus 5.56%, respectively; *p* = 0.03), and an increased BMI (30.42 ± 3.96 versus 26.74 ± 3.26 kg/sqm, *p* = 0.011) was observed. These findings suggest that in females with low ACTH + MACS, a higher rate of metabolic disturbances might be detected, and this may be indirectly reflected in the associated comorbidities, including osseous ones. Moreover, another MACS sub-analysis showed that blood Crosslaps levels were statistically significantly higher in MACS-negative subjects with low ACTH than in those with normal ACTH (0.60 ± 0.27 versus 0.42 ± 0.21 ng/mL, *p* = 0.022). This suggests that in patients with tumours that are considered true non-secretors (with a second-day plasma cortisol below 1.8 µg/dL), tumour-associated bone resorption (as indicated by the collagen-derived biomarker) might be reflected by a suppressed baseline ACTH. Furthermore, it is still debatable whether a subgroup of patients diagnosed with NFATs/MACS is prone to skeletal damage, and it seems more likely that the surrogate biomarkers or models that point out this higher risk originate from the adrenal panel. Overall, these data suggest that closer monitoring of patients with ACTH < 10 pg/mL should be performed even if they are DST-negative. Moreover, ACTH might become a tool for addressing adrenalectomy in these patients based on the spectrum of complications.

### 4.2. Pathogenic Pathways of Bone Involvement in NFATs/MACS

The analysis of even mild cortisol secretion in adrenal incidentalomas shows heterogeneous results in daily practice. The pathogenic background involves various contributors that are directly cortisol-related effects or indirectly prone to bone anomalies (as seen for cardio-metabolic complications), e.g., increased oxidative stress or chronic inflammation [44,75,76,77,78].

Cortisol acts via the glucocorticoid receptor at the bone level, and, generally, the cortisol actions involve a negative effect on bone formation with osteoblast differentiation inhibition while stimulating their apoptosis. A sclerostin increase, as well as osteocalcin and osteoprotegerin decrease elevates the activity of receptor activator of nuclear factor κβ ligand (RANKL), and, hence ostoclastogenesis is stimulated. This enhances the bone loss, in addition to the 11-β-hydroxysteroid dehydrogenase type 1–mediated osteoblast effects, damage of the bone microarchitecture, and elevation of the osteocyte apoptosis. The larger frame of glucocorticoid actions also includes the reduction of calcium intestinal absorption and renal tubular reabsorption; altered growth hormone and gonadotropins secretion, and an impairment of PTH effects on bone by interfering with the PTH receptor [77,79,80,81]. In this study, as mentioned, the mineral metabolism hormones (PTH and 25OHD), as well as total serum calcium, were similar, regardless of the subgroup.

In NFATs/MACS, DXA-based BMD might not be affected, and the increase in fracture risk may involve an anomaly of bone quality or an elevated fall risk due to muscle impairment/sarcopenia, frailty, and diabetes-associated complications. As applied in the type 2 diabetic menopausal population, TBS might prove useful, but current statistical evidence is limited. In the present study, TBS showed no differences, regardless of ACTH cut-offs ± MACS. Alternative tools such as the spinal deformity index or high-resolution peripheral quantitative computed tomography may be applied to some extent, but currently they are not recommended in everyday practice. Other tools are represented by biomarkers such as DHEA-S (dehydroepiandrosterone-sulphate) or the cortisol/DHEA-S ratio, or by integrated multifactorial algorithms that take into consideration bone turnover markers, age, menopause duration, type 2 diabetes, etc. [80,81,82,83,84].

### 4.3. The Larger (Non-Osseous) Frame in NFATs/MACS

The study population had a type 2 diabetes rate of 18%, an arterial hypertension rate of 75%, and a dyslipidemia rate of 78%. We found that MACS-positive versus MACS-negative tumours in patients with suppressed ACTH had a higher prevalence of diabetes, which was not reflected in BMD/TBS differences. For example, we mention one recent study in 70 MACS-positive versus 101 MACS-free tumours showing a higher prevalence of dyslipidemia (96% versus 52%, *p* = 0.037), but not diabetes, while a baseline ACTH level of <15 pg/mL was an independent MACS predictor [84]. Another study from 2025 also found a higher rate of diabetes in MACS-positive versus MACS-negative patients (35% versus 20%) [85].

### 4.4. Current Limits and Further Expansion

As limitations, in addition to the retrospective design of data collection and the sample size, we mention that we limited the inclusion of post-menopausal women who were not under anti-osteoporotic drugs to avoid the bias that comes from active intervention to reduce fracture risk and enhance BMD. Also, we restricted (from a technical perspective) the patients who did not have cortisol/ACTH registration times within the mentioned frame in order to avoid the bias of inadequate/incorrect testing.

Of note, we applied the term “NFATs” in both MACS-positive and MACS-negative tumours, since we addressed ACTH cut-offs, not the second-day plasma cortisol after DST, but recently, NFAT was proposed as an exclusive MACS-negative category. Notably, a large panel of exclusion criteria was applied (e.g., anti-obesity medication, bariatric surgery, adrenalectomy, etc.) to avoid the bias associated with the glucose metabolism-related influence on bone status, weight-associated anomalies of the skeletal health, and other hormonal connections.

Also, baseline 25-hydroxyvitamin D was mean of 24.9 ng/mL, which represents an insufficient level, noting that vitamin D alterations might act as a confounder for bone parameter interpretation. Other biases might be related to the potential assay variability for cortisol and ACTH (Roche ECLIA), the influence of obesity/overweight (as shown by mean BMI), which independently affects cortisol dynamics, and the fact that overall we analyzed a population with a relatively low prevalence of fractures (4.76%), which might limit fracture-specific conclusions.

Further expansion across a longitudinal design, as well as the inclusion of males and pre-menopausal women is mandatory. The retrospective design, lack of longitudinal follow-up, and the single-centre setting potentially limit generalizability to diverse populations, and further external validation is required. To the best of our knowledge, this study adds to the limited number of previous clinical studies that addressed the osseous biomarkers in the field of adrenal incidentalomas.

## 5. Conclusions

In this study of ACTH-related bone impairment in menopausal women diagnosed with adrenal incidentalomas, two-thirds of patients had a non-normal BMD at DXA, with a prevalent rate of osteoporotic fractures of almost 5%. While the lowest mean T-score showed osteopenia and lumbar DXA-derived TBS pointed a partially degraded microarchitecture, FRAX and FRAXplus estimations correlated with all three central DXA sites, regardless of the ACTH cut-off. Patients with a low ACTH displayed similar bone/adrenal features to those with normal ACTH, except for a higher MACS rate and a larger tumour. Fracture estimation showed that only in patients with low ACTH, MOF adjusted for lumbar BMD was lower than MOF adjusted for diabetes, and HF was lower when adjusted for lumbar BMD; hence, FRAXplus might be a better predictor tool. ACTH correlated with lumbar BMD only in the group with suppressed values, and this suggests its potential usefulness as a bone biomarker. On the other hand, MACS-negative subjects with low ACTH versus those with normal ACTH showed higher CrossLaps; hence, elevated bone resorption may be detected even in patients with tumours that are regarded as true non-secretor. A subgroup of patients diagnosed with NFATs/MACS might be prone to skeletal damage, and the use of biomarkers, including ACTH, might help refining this higher risk. We further reinforce the need for prospective longitudinal studies, standardized ACTH thresholds, and the integration of TBS and FRAXplus into future NFAT/MACS risk stratification.

## Figures and Tables

**Table 1:** Inclusion and exclusion criteria according to our methods.

Inclusion criteria	confirmed menopause
	age of 50 years or older
	written consent during hospitalization (inpatient)
	imaging diagnosis of an adrenal incidentaloma
	available data (mineral and adrenal metabolism assays, adrenal CT scan, central DXA) within a maximum one-month gap between these assessments
Exclusion criteria	overt (clinically manifested) Cushing’s syndrome
	Cushing’s disease, suspected/confirmed paraneoplastic Cushing’s syndrome
	active endocrine tumours
	neuroendocrine neoplasia
	multiple endocrine neoplasia syndrome
	active thyroid dysfunction [we excluded the patients who did not present a normal value of freeT4 (thyroxine) and TSH (thyroid stimulating hormone)]
	bone metabolic conditions
	active infectious or inflammatory disorders
	chronic kidney disease
	type 1 and secondary diabetes mellitus
	prior or current exposure to specific anti-osteoporotic drugs
	corticotherapy
	insulin therapy
	GLP-1 medication
	bariatric surgery
	unilateral or bilateral adrenalectomy
	clear and conclusive results of the CT (both adrenal glands), DXA (lumbar, bone mineral density, total hip), and TBS
	second-day cortisol following 1 mg DST of less than 5 µg/dL
	suspected imaging features of an adrenal malignancy (primary or secondary)
	registration time of the cortisol and ACTH assays (baseline) and second-day cortisol (DST) between 6:00 and 7:30 a.m. (for both cortisol and ACTH on the first day and for second-day cortisol)

Abbreviations: CT = computed tomography; DXA = Dual-Energy X-Ray Absorptiometry; GLP-1 = glucagon-like peptide-1 agonist; TBS = trabecular bone score.

**Table 2:** The method of parameters’ evaluation/assessment.

Parameter/Variable	Method/Manufacturer
Demographic features (age, years since menopause), comorbidities (type 2 diabetes, hypertension, dyslipidaemia), prior fragility fractures	medical records and evaluation during hospitalization
Total calcium, phosphorus, magnesium, total alkaline phosphatase, fasting glucose, creatinine	photometry (Roche)
PTH, P1NP, osteocalcin, CrossLaps, cortisol, ACTH	ECLIA (Roche)
25-hydroxyvitamin D	CLIA (DiaSorin)
Glycated hemoglobin A1c	turbidimetry (Roche)
Central (lumbar, femoral neck, total hip) DXA	GE Lunar Prodigy device
Trabecular bone score	TBS iNsight software v3.0

Abbreviations: ACTH = adrenocorticotropic hormone; CLIA = Clinical Laboratory Improvement Amendments; DXA = Dual-Energy X-Ray Absorptiometry; ECLIA = electrochemiluminescence immunoassay; PTH = parathyroid hormone; P1NP = procollagen type 1 N-terminal pro-peptide.

**Table 3:** Analysis of the demographic characteristics, baseline biochemistry panel, and comorbidities.

Parameter	Value	Normal Range
Number of patients (%)	84 (100%)	
Age (years), mean ± SD	61.49 ± 7.86	
Years since menopause, mean ± SD	14.23 ± 8.73	
Type 2 diabetes, N (%)	15 (18.07)	
Arterial hypertension, N (%)	63 (75.00)	
Dyslipidemia, N (%)	66 (78.57)	
Body mass index (kg/sqm), mean ± SD	29.48 ± 5.70	
Fasting glycemia (mg/dL), mean ± SD	108.60 ± 26.81	74–106
Glycated hemoglobin A1c (%), mean ± SD	6.13 ± 1.22	4.8–5.9
Serum creatinine (mg/dL), mean ± SD	0.79 ± 0.18	0.7–1.2
Baseline ACTH (pg/mL), M (Q1, Q3)	11.89 (7.93, 16.15)	3–66
Suppressed ACTH (group S), N (%)	33 (39.29)	
Morning plasma (baseline) cortisol (µg/dL), median (Q1, Q3)	13.01 (9.82, 14.96)	6.2–19.4
Second-day plasma cortisol after 1 mg DST (µg/dL), M (Q1, Q3)	1.68 (1.04, 2.79)	<1.8
MACS-positive, N (%)	26 (30.95)	
Largest tumour diameter (cm), mean ± SD	2.25 ± 0.99	
Prevalence of unilateral tumour, N (%)	55 (65.48)	

Abbreviations: ACTH = adrenocorticotropic hormone; DST = dexamethasone suppression test; M = median; MACS = mild autonomous cortisol secretion; Q = quartile SD = standard deviation.

**Table 4:** Mineral metabolism assays, bone turnover markers, DXA assessment, and FRAX-based probabilities.

Parameter	Value	Normal Range
**Mineral metabolism**		
Total serum calcium (mg/dL), mean ± SD	9.58 ± 0.49	8.4–10.2
Serum ionized calcium (mg/dL), mean ± SD	4.13 ± 0.28	3.9–4.9
Serum phosphorus (mg/dL), mean ± SD	3.71 ± 0.56	2.5–4.5
Serum magnesium (mg/dL), mean ± SD	1.98 ± 0.20	1.6–2.6
25-hydroxyvitamin D (ng/mL), mean ± SD	24.90 ± 11.37	30–100
PTH (pg/mL), mean ± SD	45.42 ± 15.22	15–65
**Bone turnover markers**		
Osteocalcin (ng/mL), mean ± SD	23.31 ± 11.65	14–46
Alkaline phosphatase (U/L), mean ± SD	80.46 ± 31.83	35–129
P1NP (ng/mL), mean ± SD	56.11 ± 22.87	20.25–76.31
CrossLaps (ng/mL), mean ± SD	0.49 ± 0.25	0.33–0.782
**DXA assessment**		
Prevalent fragility fractures, N (%)	4 (4.76)	
Bone impairment, N (%)	55 (65.48)	
Osteoporosis, N (%)	17 (21.99)	
Osteopenia, N (%)	38 (46.91)	
Lumbar BMD (g/sqcm), mean ± SD	1.066 ± 0.167	
Lumbar T-score (SD), mean ± SD	−1.00 ± 1.39	>−1
Femoral neck BMD (g/sqcm), mean ± SD	0.860 ± 0.145	
Femoral neck T-score (SD), mean ± SD	−1.12 ± 1.00	>−1
Total hip BMD (g/sqcm), mean ± SD	0.937 ± 0.178	
Total hip T-score (SD), mean ± SD	−0.68 ± 1.18	>−1
TBS, M (Q1, Q3)	1.320 (1.230, 1.392)	>1.350
**FRAX-based probabilities**		
MOF with femoral neck BMD (%), M (Q1, Q3)	3.90 (2.83, 6.15)	
MOF adjusted for lumbar BMD (%), M (Q1, Q3)	2.95 (2.20, 4.40)	
MOF adjusted for diabetes (%), M (Q1, Q3)	3.50 (3.00, 6.10)	
HF with femoral neck BMD (%), M (Q1, Q3)	0.70 (0.30, 1.78)	
HF adjusted for lumbar BMD (%), M (Q1, Q3)	0.50 (0.20, 1.23)	
HF adjusted for diabetes (%), M (Q1, Q3)	0.60 (0.30, 2.83)	

Abbreviations: BMD = bone mineral density; DXA = Dual-Energy X-Ray Absorptiometry; HF = 10-year hip fracture risk; M = median; MOF = 10-year major osteoporotic fracture risk; N = number of patients; PTH = parathyroid hormone; P1NP = procollagen 1 N-terminal pro-peptide; Q = quartile; SD = standard deviation; TBS = trabecular bone score.

**Table 5:** Correlations between NFAT characteristics and central DXA-based BMD and FRAX-based probabilities.

Parameter	Baseline ACTH (pg/mL)	Morning Plasma (Baseline) Cortisol (µg/dL)	Second-Day Plasma Cortisol After 1 mg DST (µg/dL)	Largest Tumour Diameter (cm)	Lumbar BMD (g/sqcm)	Femoral Neck BMD (g/sqcm)	Total Hip BMD (g/sqcm)	MOF with Femoral Neck BMD	MOF Adjusted for Lumbar BMD	MOF Adjusted for Diabetes	HF with Femoral Neck BMD	HF Adjusted for Lumbar BMD	HF Adjusted for Diabetes
Baseline ACTH (pg/mL)		**r = 0.228** ***p* = 0.039**	**r = −0.301** ***p* = 0.024**	**r = −0.434** ***p* < 0.001**	r = −0.078 *p* = 0.527	r = −0.075 *p* = 0.578	r = −0.077 *p* = 0.600	r = 0.018 *p* = 0.903	r = −0.001 *p* = 0.997	r = −0.358 *p* = 0.280	r = −0.009 *p* = 0.950	r = 0.005 *p* = 0.976	r = −0.395 *p* = 0.258
Morning plasma (baseline) cortisol (µg/dL)			r = −0.021 *p* = 0.878	r = −0.067 *p* = 0.566	r = 0.118 *p* = 0.347	r = 0.047 *p* = 0.733	r = −0.154 *p* = 0.295	r = −0.036 *p* = 0.811	r = −0.018 *p* = 0.907	r = 0.276 *p* = 0.412	r = −0.125 *p* = 0.401	r = −0.075 *p* = 0.625	r = 0.363 *p* = 0.303
Second-day plasma cortisol after 1 mg DST (µg/dL)				**r = 0.572** ***p* < 0.001**	r = 0.109 *p* = 0.461	r = 0.134 *p* = 0.428	r = 0.110 *p* = 0.500	r = −0.102 *p* = 0.591	r = −0.204 *p* = 0.287	r = 0.288 *p* = 0.452	r = −0.090 *p* = 0.635	r = −0.123 *p* = 0.526	r = 0.286 *p* = 0.493
Largest tumour diameter (cm)					r = 0.012 *p* = 0.923	r = 0.048 *p* = 0.739	r = 0.191 *p* = 0.209	r = 0.010 *p* = 0.951	r = −0.017 *p* = 0.913	r = −0.133 *p* = 0.696	r = −0.009 *p* = 0.952	r = −0.058 *p* = 0.714	r = −0.143 *p* = 0.693

Abbreviations: ACTH = adrenocorticotropic hormone; BMD = bone mineral density; DST = dexamethasone suppression test; HF = 10-year hip fracture risk; MOF = 10-year major osteoporotic fracture risk.

**Table 6:** Correlations between central DXA-based BMD and FRAX-based estimation of fracture risk.

Parameter	MOF with Femoral Neck BMD	MOF Adjusted for Lumbar BMD	MOF Adjusted for Diabetes	HF with Femoral Neck BMD	HF Adjusted for Lumbar BMD	HF Adjusted for Diabetes
Lumbar BMD (g/sqcm)	**r = −0.510** ***p* < 0.001**	**r = −0.505** ***p* < 0.001**	r = 0.031 *p* = 0.933	**r = −0.463** ***p* < 0.001**	**r = −0.475** ***p* < 0.001**	r = 0.050 *p* = 0.898
Femoral neck BMD (g/sqcm)	**r = −0.878** ***p* < 0.001**	**r = −0.811** ***p* < 0.001**	r = −0.460 *p* = 0.181	**r = −0.925** ***p* < 0.001**	**r = −0.927** ***p* < 0.001**	r = −0.544 *p* = 0.130
Total hip BMD (g/sqcm)	**r = −0.852** ***p* < 0.001**	**r = −0.811** ***p* < 0.001**	r = −0.741 *p* = 0.057	**r = −0.862** ***p* < 0.001**	**r = −0.876** ***p* < 0.001**	r = −0.600 *p* = 0.208

Abbreviations: BMD = bone mineral density; HF = 10-year hip fracture risk; MOF = 10-year major osteoporotic fracture risk.

**Table 7:** Distribution of patients according to a 5-year age sub-category within group S and group nonS.

Age Group (years) N (%)	Group S (N = 33, 39.29%)	Group nonS (N = 51, 60.71%)	*p*-Value
45–49	5 (15.15)	1 (1.96)	0.146
50–54	3 (9.09)	4 (7.84)	
55–59	6 (18.18)	14 (27.45)	
60–64	10 (30.30)	17 (33.33)	
65–69	7 (21.21)	6 (11.76)	
70–74	0 (0.00)	3 (5.88)	
75–79	2 (6.06)	6 (11.76)	

Abbreviations: N = number of patients; red font = group S; blue font = group nonS.

**Table 8:** Demographic features, comorbidities, and adrenal panel assessment in group S versus nonS.

Parameter	Group S (N = 33, 39.29%)	Group nonS (N = 51, 60.71%)	*p*-Value
Age (years), mean ± SD	60.06 ± 8.63	62.41 ± 7.26	0.182
Years since menopause, mean ± SD	13.28 ± 8.72	14.91 ± 8.80	0.479
Type 2 diabetes mellitus, N (%)	7 (21.21)	8 (16.00)	0.572
Arterial hypertension, N (%)	25 (75.76)	38 (74.51)	0.897
Dyslipidaemia, N (%)	25 (75.76)	41 (80.39)	0.613
Body mass index (kg/sqm), mean ± SD	28.26 ± 3.96	30.46 ± 6.68	0.104
Fasting glycaemia (mg/dL), mean ± SD	106.94 ± 28.34	109.68 ± 26.14	0.712
Glycated haemoglobin A1c (%), mean ± SD	6.02 ± 0.93	6.21 ± 1.40	0.574
Serum creatinine (mg/dL), mean ± SD	0.75 ± 0.14	0.81 ± 0.21	0.276
Baseline ACTH (pg/mL), M (Q1, Q3)	7.37 (6.19, 8.60)	15.37 (12.30, 20.48)	**<0.001**
Morning plasma (baseline) cortisol (µg/dL), M (Q1, Q3)	12.27 (9.48, 14.30)	13.43 (10.01, 15.97)	0.163
Second-day plasma cortisol after 1-mg DST (µg/dL), M (Q1, Q3)	2.08 (1.58, 3.08)	1.25 (0.94, 2.02)	**0.014**
MACS, N (%)	15 (45.45)	11 (21.57)	**0.021**
Largest tumour diameter (cm), mean ± SD	2.67 ± 0.98	1.98 ± 0.92	**0.003**
Unilateral tumour, N (%)	20 (60.61)	35 (68.63)	0.450

Abbreviations: ACTH = adrenocorticotropic hormone; DST = dexamethasone suppression test; M = median; MACS = mild autonomous cortisol secretion; N = number of patients; Q = quartile; SD = standard deviation; red font = group S; blue font = group nonS.

**Table 9:** Sensitivity and specificity of the 2.45 cm cut-off value for the largest tumour diameter, respectively, of 0.32 ng/mL cut-off value for CrossLaps for predicting suppressed ACTH (<10 pg/mL).

**Cut-off Value Largest Tumour Diameter (cm) for Suppressed ACTH**	**AUC**	**Sensitivity (95% CI)**	**Specificity (95% CI)**	**Youden Index**
2.45 cm	0.702	63.30 (45.51–78.13)	75.00 (61.22–85.08)	0.383
**Cut-off value of CrossLaps (ng/mL) for suppressed ACTH**	**AUC**	**Sensitivity (95% CI)**	**Specificity (95% CI)**	**Youden Index**
0.32 ng/mL	0.647	87.50 (69.00–95.66)	39.50 (25.60–55.28)	0.270

Abbreviations: ACTH = adrenocorticotropic hormone; AUC = area under curve; CI = confidence interval.

**Table 10:** Mineral metabolism assays, bone turnover markers, DXA assessment and FRAX-based probabilities in group S and group nonS.

Parameter	Group S (N = 33, 39.29%)	Group nonS (N = 51, 60.71%)	*p*-Value
**Mineral metabolism**			
Total serum calcium (mg/dL), mean ± SD	9.58 ± 0.49	9.58 ± 0.49	0.990
Serum ionized calcium (mg/dL), mean ± SD	4.19 ± 0.23	4.09 ± 0.31	0.191
Serum phosphorus (mg/dL), mean ± SD	3.72 ± 0.64	3.70 ± 0.50	0.866
Serum magnesium (mg/dL), mean ± SD	1.99 ± 0.24	1.96 ± 0.17	0.557
25-hydroxyvitamin D (ng/mL), mean ± SD	23.03 ± 10.07	26.21 ± 12.16	0.258
PTH (pg/mL), mean ± SD	41.85 ± 13.81	47.84 ± 15.86	0.165
**Bone turnover markers**			
Osteocalcin (ng/mL), mean ± SD	25.33 ± 14.16	22.05 ± 9.77	0.294
Alkaline phosphatase (U/L), mean ± SD	91.15 ± 38.84	73.49 ± 24.30	**0.017**
P1NP (ng/mL), mean ± SD	58.35 ± 29.32	55.02 ± 19.54	0.661
CrossLaps (ng/mL), mean ± SD	0.56 ± 0.29	0.44 ± 0.22	**0.050**
**DXA assessment**			
Prevalent fragility fractures, N (%)	3 (9.09)	1 (1.96)	0.295
Bone impairment, N (%)	22 (66.67)	33 (64.71)	0.853
Osteoporosis, N (%)	8 (24.24)	9 (18.75)	0.551
Osteopenia, N (%)	14 (42.42)	24 (50.00)	0.651
Lumbar BMD (g/sqcm), mean ± SD	1.087 ± 0.173	1.052 ± 0.163	0.404
Lumbar T-score (SD), mean ± SD	−0.85 ± 1.49	−1.10 ± 1.33	0.453
Femoral neck BMD (g/sqcm), mean ± SD	0.875 ± 0.155	0.851 ± 0.141	0.557
Femoral neck T-score (SD), mean ± SD	−1.02 ± 1.17	−1.18 ± 0.88	0.573
Total hip BMD (g/sqcm), mean ± SD	0.930 ± 0.185	0.940 ± 0.177	0.846
Total hip T-score (SD), mean ± SD	−0.75 ± 1.40	−0.63 ± 1.05	0.719
TBS, M (Q1, Q3)	1.337 (1.244, 1.429)	1.291 (1.199, 1.401)	0.393
**FRAX-based probabilities**			
MOF with femoral neck BMD (%), M (Q1, Q3)	3.60 (2.80, 6.15)	3.95 (2.93, 6.15)	0.630
MOF adjusted for lumbar BMD (%), M (Q1, Q3)	2.80 (2.10, 4.23)	3.05 (2.30, 4.40)	0.471
MOF adjusted for diabetes (%), M (Q1, Q3)	4.40 (3.38, 6.60)	3.50 (2.00, 7.55)	0.461
HF with femoral neck BMD (%), M (Q1, Q3)	0.85 (0.20, 1.95)	0.65 (0.30, 1.68)	0.801
HF adjusted for lumbar BMD (%), M (Q1, Q3)	0.65 (0.18, 1.00)	0.45 (0.23, 1.35)	0.520
HF adjusted for diabetes (%), M (Q1, Q3)	2.10 (0.40, 2.95)	0.40 (0.20, 3.60)	0.402

Abbreviations: BMD = bone mineral density; DXA = Dual-Energy X-Ray Absorptiometry; HF = 10-year hip fracture risk; M = median; MOF = 10-year major osteoporotic fracture risk; M = median; N = number of patients; PTH = parathormone; P1NP = procollagen 1 N-terminal pro-peptide; Q = quartile; SD = standard deviation; TBS = trabecular bone score; red font = group S; blue font = group nonS.

**Table 11:** FRAX-based probabilities for MOF and HF within group S and nonS.

	**MOF with Femoral Neck BMD-MOF Adjusted for Lumbar BMD** ***p*-Value**	**MOF Adjusted for Lumbar BMD-MOF Adjusted for Diabetes** ***p*-Value**	**MOF Adjusted for Diabetes-MOF Adjusted for Lumbar BMD** ***p*-Value**
Group S	**<0.001**	0.832	**0.036**
Group nonS	**<0.001**	0.289	0.244
	**HF with femoral neck BMD-HF adjusted for lumbar BMD** ***p*-value**	**HF adjusted for lumbar BMD-HF adjusted for diabetes** ***p*-value**	**HF adjusted for diabetes-HF adjusted for lumbar BMD** ***p*-value**
Group S	**0.007**	0.068	0.095
Group nonS	0.111	0.439	0.300

Abbreviations: BMD = bone mineral density; HF = 10-year hip fracture risk; MOF = 10-year major osteoporotic fracture risk; red font = group S; blue font = group nonS.

**Table 12:** Correlations between NFAT’s (hormonal and CT) characteristics with central DXA-BMD, respectively, FRAX-based calculations within group S and group nonS.

	Baseline ACTH (pg/mL)	Morning Plasma (Baseline) Cortisol (µg/dL)	Second-Day Plasma Cortisol After 1-mg DST (µg/dL)	Largest Tumour Diameter (cm)	Lumbar BMD (g/sqcm)	Femoral Neck BMD (g/sqcm)	Total Hip BMD (g/sqcm)	MOF with Femoral Neck BMD	MOF Adjusted for Lumbar BMD	HF with Femoral Neck BMD	HF Adjusted for Lumbar BMD
**Group S**											
Baseline ACTH (pg/mL)		r = −0.082 *p* = 0.649	r = 0.016 *p* = 0.942	r = −0.095 *p* = 0.942	**r = 0.434** ***p* = 0.024**	r = 0.149 *p* = 0.510	r = −0.067 *p* = 0.791	r = 0.059 *p* = 0.803	r = −0.004 *p* = 0.987	r = 0.070 *p* = 0.769	r = −0.087 *p* = 0.730
Morning plasma (baseline) cortisol (µg/dL)	r = −0.082 *p* = 0.649		r = 0.170 *p* = 0.427	r = 0.273 *p* = 0.145	r = 0.250 *p* = 0.208	r = 0.204 *p* = 0.363	r = 0.251 *p* = 0.316	r = −0.056 *p* = 0.816	r = −0.066 *p* = 0.794	r = −0.245 *p* = 0.299	r = −0.306 *p* = 0.217
Second-day plasma cortisol after 1-mg DST (µg/dL)	r = 0.016 *p* = 0.942	r = 0.170 *p* = 0.427		**r = 0.591** ***p* = 0.002**	r = 0.049 *p* = 0.842	r = 0.176 *p* = 0.547	r = 0.136 *p* = 0.630	r = −0.149 *p* = 0.628	r = −0.161 *p* = 0.617	r = −0.085 *p* = 0.781	r = −0.165 *p* = 0.608
Largest tumor diameter (cm)	r = −0.095 *p* = 0.942	r = 0.273 *p* = 0.145	**r = 0.591** ***p* = 0.002**		r = 0.027 *p* = 0.897	r = 0.052 *p* = 0.833	r = 0.093 *p* = 0.722	r = −0.042 *p* = 0.874	r = −0.048 *p* = 0.860	r = −0.153 *p* = 0.557	r = −0.245 *p* = 0.360
**Group nonS**											
Baseline ACTH (pg/mL)		**r = 0.315** ***p* = 0.028**	r = −0.146 *p* = 0.426	**r = −0.395** ***p* = 0.005**	r = −0.185 *p* = 0.246	r = −0.172 *p* = 0.322	r = −0.229 *p* = 0.214	r = −0.177 *p* = 0.368	r = −0.252 *p* = 0.196	r = −0.195 *p* = 0.320	r = −0.165 *p* = 0.402
Morning plasma (baseline) cortisol (µg/dL)	**r = 0.315** ***p* = 0.028**		r = 0.081 *p* = 0.664	r = −0.223 *p* = 0.137	r = 0.058 *p* = 0.724	r = −0.055 *p* = 0.759	**r = −0.391** ***p* = 0.033**	r = −0.020 *p* = 0.923	r = −0.034 *p* = 0.868	r = −0.108 *p* = 0.593	r = −0.027 *p* = 0.893
Second-day plasma cortisol after 1-mg DST (µg/dL)	r = −0.146 *p* = 0.426	r = 0.081 *p* = 0.664		r = 0.339 *p* = 0.067	r = 0.149 *p* = 0.440	r = 0.238 *p* = 0.274	r = 0.120 *p* = 0.566	r = −0.058 *p* = 0.826	r = −0.089 *p* = 0.736	r = −0.202 *p* = 0.437	r = −0.097 *p* = 0.710
Largest tumor diameter (cm)	**r = −0.395** ***p* = 0.005**	r = −0.223 *p* = 0.137	r = 0.339 *p* = 0.067		r = −0.073 *p* = 0.664	r = −0.040 *p* = 0.829	r = 0.167 *p* = 0.395	r = 0.225 *p* = 0.268	r = 0.258 *p* = 0.204	r = 0.245 *p* = 0.227	r = 0.203 *p* = 0.319

Abbreviations: ACTH = adrenocorticotropic hormone; BMD = bone mineral density; DST = dexamethasone suppression test; HF = 10- year hip fracture risk; MOF = 10-year major osteoporotic fracture risk; red font = group S; blue font = group nonS.

**Table 13:** Correlations between DXA-BMD and FRAX-based probabilities within group S and group nonS.

Parameter	MOF with Femoral Neck BMD	MOF Adjusted for Lumbar BMD	HF with Femoral Neck BMD	HF Adjusted for Lumbar BMD
**Group S**				
Lumbar BMD (g/sqcm)	**r = −0.537** ***p* = 0.021**	**r = −0.526** ***p* = 0.025**	**r = −0.523** ***p* = 0.026**	**r = −0.494** ***p* = 0.037**
Femoral neck BMD (g/sqcm)	**r = −0.817** ***p* < 0.001**	**r = −0.694** ***p* = 0.001**	**r = −0.943** ***p* < 0.001**	**r = −0.919** ***p* < 0.001**
Total hip BMD (g/sqcm)	**r = −0.760** ***p* = 0.004**	**r = −0.709** ***p* = 0.015**	**r = −0.823** ***p* = 0.001**	**r = −0.858** ***p* < 0.001**
**Group nonS**				
Lumbar BMD (g/sqcm)	**r = −0.499** ***p* = 0.007**	**r = −0.503** ***p* = 0.006**	**r = −0.489** ***p* = 0.008**	**r = −0.490** ***p* = 0.008**
Femoral neck BMD (g/sqcm)	**r = −0.911** ***p* < 0.001**	**r = −0.862** ***p* < 0.001**	**r = −0.894** ***p* < 0.001**	**r = −0.935** ***p* < 0.001**
Total hip BMD (g/sqcm)	**r = −0.869** ***p* < 0.001**	**r = −0.842** ***p* < 0.001**	**r = −0.770** ***p* < 0.001**	**r = −0.841** ***p* < 0.001**

Abbreviations: BMD = bone mineral density; HF = 10-year hip fracture risk; MOF = 10-year major osteoporotic fracture risk; red font = group S; blue font = group nonS.

**Table 14:** Multiple linear regression model to predict baseline ACTH.

	Baseline ACTH (pg/mL)		
Parameter	B ± SE	β	*p*-Value
Constant	13.65 ± 7.32		0.079
Morning plasma (baseline) cortisol (µg/dL)	0.06 ± 0.16	0.05	0.710
MACS	−1.23 ± 2.13	−0.08	0.567
**Largest tumour diameter (cm)**	**−3.07 ± 0.98**	**−0.43**	**0.003**
Age (years)	−0.01 ± 0.11	−0.02	0.905
BMI (kg/sqm)	0.21 ± 0.15	0.18	0.165
Model summary	**R Square = 0.225**		**0.016**

Abbreviations: ACTH = adrenocorticotropic hormone; BMI = body mass index; MACS = mild autonomous cortisol secretion; B = unstandardized regression coefficient; SE = standard error; β = standardized regression coefficient; R Square = coefficient of determination.

**Table 15:** Demographic features, panel of analysed comorbidities, and adrenal (hormonal and CT) profile in group S versus nonS: MACS sub-analysis.

	Group S			Group nonS				
Parameter	MACS (N = 15, 45.45%)	nonMACS (N = 18, 54.55%)	*p* -Value	MACS (N = 11, 21.57%)	nonMACS (N = 40, 78.43%)	*p* -Value	p1-Value	p2-Value
Age (years), mean ± SD	57.80 ± 7.61	61.94 ± 9.18	0.173	63.45 ± 4.63	62.13 ± 7.85	0.483	0.701	0.943
Years since menopause, mean ± SD	48.44 ± 4.95	48.81 ± 3.69	0.178	49.29 ± 3.09	48.75 ± 3.60	0.934	0.254	0.973
Type 2 diabetes mellitus, N (%)	6 (40.00)	1 (5.56)	**0.030**	2 (18.18)	6 (15.38)	0.999	0.395	0.413
Arterial hypertension, N (%)	12 (80.00)	13 (72.22)	0.699	9 (81.82)	29 (72.50)	0.706	0.907	0.983
Dyslipidaemia, N (%)	12 (80.00)	13 (72.00)	0.699	10 (90.91)	31 (77.50)	0.428	0.614	0.744
BMI (kg/sqm), mean ± SD	30.42 ± 3.96	26.74 ± 3.26	**0.011**	34.41 ± 5.31	29.67 ± 6.71	0.091	0.090	0.098
Fasting glycaemia (mg/dL), mean ± SD	109 ± 28.76	105.51 ± 29.14	0.784	112.00 ± 34.59	109.08 ± 24.27	0.839	0.852	0.685
Glycated haemoglobin A1c (%), mean ± SD	6.15 ± 0.68	5.87 ± 1.80	0.493	6.51 ± 1.97	6.09 ± 1.12	0.560	0.556	0.609
Serum creatinine (mg/dL), mean ± SD	0.78 ± 0.17	0.74 ± 0.13	0.478	0.89 ± 0.27	0.78 ± 0.18	0.297	0.314	0.409
Baseline ACTH (pg/mL), M (Q1, Q3)	8.00 (6.64, 8.90)	7.24 (5.87, 8.01)	0.229	14.71 (12.29, 19.51)	15.65 (12.31, 20.59)	0.801	**<0.001**	**<0.001**
Morning plasma (baseline) cortisol (μg/dL), M (Q1, Q3)	11.58 (8.77, 13.25)	13.49 (9.63, 15.12)	0.178	12.15 (9.96, 15.92)	13.92 (9.90, 16.66)	0.455	0.605	0.668
Second-day plasma cortisol after 1 mg DST (μg/dL), M (Q1, Q3)	2.79 (2.18, 3.44)	1.34 (1.01, 1.64)	**<0.001**	2.80 (1.88, 4.86)	1.02 (0.83, 1.25)	**<0.001**	0.919	0.056
Largest tumour diameter (cm), mean ± SD	3.12 ± 0.74	2.21 ± 0.99	**0.008**	2.51 ± 0.96	1.84 ± 0.87	**0.039**	0.087	0.888

Abbreviations: BMI = body mass index; MACS = mild autonomous cortisol secretion; SD = standard deviation; red font = group S; blue font = group nonS; p1-value between MACS from group S and MACS from group nonS; p2-value between nonMACS from group S and nonMACS from group nonS.

**Table 16:** Mineral metabolism assays, bone turnover markers, DXA, and FRAX/FRAXplus results in MACS versus nonMACS within group S and nonS.

	Group S			Group nonS				
Parameter	MACS (N = 15, 45.45%)	nonMACS (N = 18, 54.55%)	*p* -Value	MACS (N = 11, 21.57%)	nonMACS (N = 40, 78.43%)	*p* -Value	p1-Value	p2-Value
**Mineral metabolism**								
Total serum calcium (mg/dL), mean ± SD	9.49 ± 0.47	9.65 ± 0.51	0.343	9.54 ± 0.30	0.59 ± 0.53	0.735	0.731	0.650
Serum ionized calcium (mg/dL), mean ± SD	4.11 ± 0.20	4.26 ± 0.24	0.105	4.19 ± 0.25	4.06 ± 0.33	0.208	0.404	0.054
Serum phosphorus (mg/dL), mean ± SD	3.82 ± 0.62	3.64 ± 0.67	0.455	3.59 ± 0.37	3.73 ± 0.53	0.361	0.314	0.609
Serum magnesium (mg/dL), mean ± SD	1.90 ± 0.26	2.06 ± 0.22	0.069	1.95 ± 0.21	1.97 ± 0.17	0.746	0.651	0.088
25-hydroxyvitamin D (ng/mL), mean ± SD	24.95 ± 10.60	21.36 ± 9.64	0.356	25.63 ± 13.63	26.38 ± 11.93	0.883	0.897	0.163
PTH (pg/mL), mean ± SD	39.86 ± 10.87	42.84 ± 15.35	0.653	45.66 ± 10.95	48.48 ± 17.17	0.686	0.340	0.317
**Bone turnover markers**								
Osteocalcin (ng/mL), mean ± SD	23.90 ± 12.63	26.24 ± 15.46	0.708	23.09 ± 12.73	21.77 ± 9.05	0.790	0.897	0.238
Alkaline phosphatase (U/L), mean ± SD	82.14 ± 19.97	99.03 ± 49.29	0.223	71.10 ± 30.64	74.14 ± 22.71	0.775	0.296	0.069
P1NP (ng/mL), mean ± SD	51.28 ± 36.02	65.41 ± 21.18	0.388	52.14 ± 17.74	55.94 ± 20.38	0.643	0.956	0.298
CrossLaps (ng/mL), mean ± SD	0.51 ± 0.32	0.60 ± 0.27	0.469	0.49 ± 0.27	0.42 ± 0.21	0.473	0.922	**0.022**
**DXA assessment**								
Prevalent fragility fractures, N (%)	1 (6.67)	2 (11.11)	0.999	0 (0.00)	1 (2.50)	0.999	0.382	0.225
Bone impairment, N (%)	9 (60.00)	13 (72.22)	0.488	7 (63.64)	26 (65.00)	0.933	0.851	0.764
Osteoporosis, N (%)	3 (20.00)	5 (27.78)	0.699	2 (18.18)	7 (18.92)	0.956	0.907	0.499
Osteopenia, N (%)	6 (40.00)	8 (44.44)	0.999	5 (45.45)	19 (51.35)	0.731	0.781	0.775
Lumbar BMD (g/sqcm), mean ± SD	1.100 ± 0.168	1.078 ± 0.181	0.747	1.079 ± 0.103	1.044 ± 0.179	0.445	0.733	0.539
Lumbar T-score (SD), mean ± SD	−0.65 ± 1.23	−1.02 ± 1.69	0.516	−0.79 ± 1.23	−1.19 ± 1.36	0.370	0.777	0.682
Femoral neck BMD (g/sqcm), mean ± SD	0.893 ± 0.146	0.860 ± 0.166	0.635	0.915 ± 0.085	0.829 ± 0.150	0.118	0.694	0.575
Femoral neck T-score (SD), mean ± SD	−0.78 ± 1.19	−1.23 ± 1.16	0.388	−0.78 ± 0.60	−1.32 ± 0.93	0.115	0.996	0.798
Total hip BMD (g/sqcm), mean ± SD	0.961 ± 0.192	0.905 ± 0.185	0.540	0.994 ± 0.083	0.922 ± 0.198	0.332	0.671	0.821
Total hip T-score (SD), mean ± SD	−0.53 ± 1.27	−0.97 ± 1.55	0.470	−0.18 ± 0.70	−0.79 ± 1.11	0.134	0.447	0.677
TBS, median (Q1, Q3)	1.354 (1.259, 1.517)	1.336 (1.229, 1.429)	0.730	1.350 (1.341, 1.358)	1.280 (1.177, 1.444)	0.410	0.999	0.583
**FRAX-based probabilities**								
MOF with femoral neck BMD (%), M (Q1, Q3)	3.10 (2.00, 4.10)	4.90 (3.20, 8.70)	0.080	3.10 (2.70, 4.45)	4.20 (2.90, 6.20)	0.318	0.699	0.772
MOF adjusted for lumbar BMD (%), M (Q1, Q3)	2.15 (1.50, 2.68)	3.70 (2.73, 6.30)	**0.021**	2.40 (2.20, 3.30)	3.10 (2.30, 4.50)	0.264	0.354	0.603
HF with femoral neck BMD (%), M (Q1, Q3)	0.30 (0.15, 1.55)	0.90 (0.30, 2.10)	0.370	0.30 (0.20, 0.70)	0.70 (0.40, 1.90)	0.107	0.898	0.942
HF adjusted for lumbar BMD (%), M (Q1, Q3)	0.20 (0.10, 0.78)	0.70 (0.20, 1.80)	0.146	0.35 (0.15, 0.55)	0.50 (0.30, 1.40)	0.193	0.833	0.893

Abbreviations: BMD = bone mineral density; DXA = Dual-Energy X-Ray Absorptiometry; HF = 10- year hip fracture risk; M = median; MOF = 10-year major osteoporotic fracture risk; PTH = parathormone; P1NP = procollagen 1 N-terminal pro-peptide; Q = quartile; SD = standard deviation; TBS = trabecular bone score; red font = group S; blue font = group nonS; p1-value between MACS from group S and MACS from group nonS; p2-value between nonMACS from group S and nonMACS from group nonS.

## Data Availability

All available data are within the manuscript.

## Abbreviations

The following abbreviations are used in this manuscript:

ACTH	adrenocorticotropic hormone
AUC	area under the curve
BMI	body mass index
BMD	bone mineral density
CT	computed tomography
CI	confidence interval
CLIA	Clinical Laboratory Improvement Amendments
DST	dexamethasone suppression test
DHEA-S	dehydroepiandrosterone-sulphate
DXA	Dual-Energy X-Ray Absorptiometry
ECLIA	electrochemiluminescence immunoassay
FRAX	Fracture Risk Assessment Tool
GLP-1	glucagon-like peptide-1 agonist
HF	10-year hip fracture risk
LTD	largest tumour diameter
MOF	10-year major osteoporotic fracture risk
MACS	mild autonomous cortisol secretion
M	median
N	number of patients
NFAT	non-functioning adrenal tumour
PTH	parathyroid hormone
P1NP	procollagen type 1 N-terminal pro-peptide
ROC	receiver operating characteristic
RANKL	receptor activator of nuclear factor κβ ligand
Q	quartile
SD	standard deviation
SOp	secondary osteoporosis
TBS	trabecular bone score
WHO	World Health Organization

## References

[1] Hofbauer L.C., Compston J.E., Saag K.G., Rauner M., Tsourdi E. (2025). Glucocorticoid-induced osteoporosis: Novel concepts and clinical implications. Lancet Diabetes Endocrinol..

[2] Gregson C.L., Armstrong D.J., Avgerinou C., Bowden J., Cooper C., Douglas L., Edwards J., Gittoes N.J.L., Harvey N.C., Kanis J.A. (2025). National Osteoporosis Guideline Group (NOGG). The 2024 UK clinical guideline for the prevention and treatment of osteoporosis. Arch. Osteoporos..

[3] Dumitru N., Carsote M., Cocolos A., Petrova E., Olaru M., Dumitrache C., Ghemigian A. (2019). The Link Between Bone Osteocalcin and Energy Metabolism in a Group of Postmenopausal Women. Curr. Health Sci. J..

[4] Mitrica M., Vasiliu O., Plesa A., Sirbu O.M. (2025). Multinodular and vacuolating neuronal tumor. Rom. J. Mil. Med..

[5] Valea A., Ghervan C., Morar A., Pop D.D., Carsote M., Albu S.E., Georgescu C.E., Chiorean A. (2016). Hashimoto’s thyroiditis and breast cancer: Coincidence or correlation?. Arch. Balk. Med. Union..

[6] Pipernea R., Popa F.L., Ciortea V.M., Irsay L., Ungur R.A., Pintea A.L., Iliescu M.G., Cipăian R.C., Stanciu M. (2023). The role of rehabilitation and anabolic treatment in severe osteoporosis associated with significant vitamin D deficiency-case report. Balneo PRM Res. J..

[7] Nistor C.E., Bugala N.M., Daguci C., Daguci L., Diaconu O.A., Rica A.M. (2023). Multiple endocrine neoplasia type 2 syndrome and osteoporosis. Aging Clin. Exp. Res..

[8] Carsote M., Valea A., Dumitru N., Terzea D., Petrova E., Albu S., Buruiana A., Ghemigian A. (2016). Metastases in daily endocrine practice. Arch. Balk. Med. Union..

[9] Popa F.L., Boicean L.C., Iliescu M.G., Stanciu M. (2021). The importance of association between sex steroids deficiency, reduction of bone mineral density and falling risk in men with implications in medical rehabilitation. Balneo PRM Res. J..

[10] Valea A., Carsote M., Moldovan C., Georgescu C. (2018). Chronic autoimmune thyroiditis and obesity. Arch. Balk. Med. Union..

[11] Owei L., Wachtel H. (2025). The Landmark Series: Evaluation and Management of Adrenal Incidentalomas. Ann. Surg. Oncol..

[12] Grazzini G., Pradella S., De Litteris F., Galluzzo A., Anichini M., Treballi F., Bicci E., Miele V. (2025). Adrenal Mass Evaluation: Suspicious Radiological Signs of Malignancy. Cancers.

[13] Zhang X., Si Y., Shi X., Zhang Y., Yang L., Yang J., Zhang Y., Leng J., Hu P., Liu H. (2025). Differentiation of multiple adrenal adenoma subtypes based on a radiomics and clinico-radiological model: A dual-center study. BMC Med. Imaging.

[14] Akkus G., Aksoydan U.P., Odabas F., Binokay H., Sert M., Tetiker T. (2025). Clinical Outcomes of Patients with Adrenal Incidentaloma-Hypertension being a Continuous Risk Factor for the Presence of Comorbidity: A Single Center’s Eight-year Experience. Curr. Med. Imaging.

[15] Montalvão P.V.G., Mangueira I.M., Alves G.D.M., Cordeiro J.V.F., Costa M.H.S., Ravanini G.A.G. (2025). Evaluation of adrenal tumors and analysis of the metabolic profile of patients with incidentaloma. Rev. Col. Bras. Cir..

[16] Boyraz A., Candemir B., Akın Ş., Candemir M., Gülçelik N.E. (2025). Increased cardiovascular risk despite unchanged body composition in non-functional adrenal incidentaloma. Ann. Endocrinol.

[17] Nistor C.E., Găvan C.S., Ciritel A.A., Nemes A.F., Ciuche A. (2022). The Association of Minimally Invasive Surgical Approaches and Mortality in Patients with Malignant Pleuropericarditis-A 10 Year Retrospective Observational Study. Medicina.

[18] Siemińska L., Siemińska K., Marek B., Kos-Kudła B., Nowak M., Głogowska-Szeląg J., Kajdaniuk D. (2024). Adrenal tumours and subclinical adrenal hyperfunction. Endokrynol. Pol..

[19] Nica S., Sionel R., Maciuca R., Csutak O., Ciobica M.L., Nica M.I., Chelu I., Radu I., Toma M. (2025). Gender-Dependent Associations Between Digit Ratio and Genetic Polymorphisms, BMI, and Reproductive Factors. Rom. J. Mil. Med..

[20] Preda E.M., Constantin N., Dragosloveanu S., Cergan R., Scheau C. (2024). An MRI-Based Method for the Morphologic Assessment of the Anterior Tibial Tuberosity. J. Clin. Med..

[21] Yoshida Y., Horiuchi K., Otsuki M., Okamoto T. (2024). Diagnosis and management of adrenal incidentaloma: Use of clinical judgment and evidence in dialog with the patient. Surg. Today.

[22] Favero V., Parazzoli C., Bernasconi D.P., Chiodini I. (2024). Cardiometabolic comorbidities and cardiovascular events in “non-functioning” adrenal incidentalomas: A systematic review and meta-analysis. J. Endocrinol. Investig..

[23] Pelsma I.C.M., Fassnacht M., Tsagarakis S., Terzolo M., Tabarin A., Sahdev A., Newell-Price J., Marina L., Lorenz K., Bancos I. (2023). Comorbidities in mild autonomous cortisol secretion and the effect of treatment: Systematic review and meta-analysis. Eur. J. Endocrinol..

[24] Araujo-Castro M., Pascual-Corrales E., Lamas C. (2023). Possible, probable, and certain hypercortisolism: A continuum in the risk of comorbidity. Ann. Endocrinol.

[25] Savoie P.H., Murez T., Neuville P., Van Hove A., Rocher L., Fléchon A., Camparo P., Ferretti L., Branger N., Rouprêt M. (2022). French AFU Cancer Committee Guidelines Update 2022–2024: Adrenal tumor-Assessment of an adrenal incidetaloma and oncological management. Prog. Urol..

[26] Braun L.T., Vogel F., Zopp S., Marchant Seiter T., Rubinstein G., Berr C.M., Künzel H., Beuschlein F., Reincke M. (2022). Whom Should We Screen for Cushing Syndrome? The Endocrine Society Practice Guideline Recommendations 2008 Revisited. J. Clin. Endocrinol. Metab..

[27] Park S.S., Kim J.H. (2023). Recent Updates on the Management of Adrenal Incidentalomas. Endocrinol Metab..

[28] Wickramarachchi B.N., Meyer-Rochow G.Y., McAnulty K., Conaglen J.V., Elston M.S. (2016). Adherence to adrenal incidentaloma guidelines is influenced by radiology report recommendations. ANZ J. Surg..

[29] Ruiz A., Michalopoulou T., Megia A., Näf S., Simón-Muela I., Solano E., Martínez L., Vendrell J. (2019). Accuracy of new recommendations for adrenal incidentalomas in the evaluation of excessive cortisol secretion and follow-up. Eur. J. Clin. Investig..

[30] Bourdeau I., El Ghorayeb N., Gagnon N., Lacroix A. (2018). Management of endocrine disease: Differential diagnosis, investigation and therapy of bilateral adrenal incidentalomas. Eur. J. Endocrinol..

[31] Capalbo M.S., Deligiannis N., Danilowicz K. (2025). Mild autonomic cortisol secretion: Comorbidities and surgical treatment outcomes. Medicina.

[32] Dragosloveanu S., Capitanu B.S., Moise M.N., Vulpe D.E., Josanu R., Gherghe M.E., Preda E.M., Cergan R., Scheau C. (2025). Restoring Hip Symmetry and Its Impact on Outcomes: A Case Series on Megaprosthesis Use in Non-Oncological Patients with Complications After Total Hip Arthroplasty. Symmetry.

[33] Katabami T., Asai S., Matsuba R., Sone M., Izawa S., Ichijo T., Tsuiki M., Okamura S., Yoshimoto T., Otsuki M. (2025). Changes in clinical features of adrenal Cushing syndrome: A national registry study. Endocr. Connect..

[34] Nistor C.E., Gavan C.S., Pantile D., Tanase N.V., Ciuche A. (2022). Cervico-Thoracic Air Collections in COVID-19 Pneumonia Patients-Our Experience and Brief Review. Chirurgia.

[35] Braun L.T., Vogel F., Nowak E., Rubinstein G., Zopp S., Ritzel K., Beuschlein F., Reincke M. (2024). Frequency of clinical signs in patients with Cushing’s syndrome and mild autonomous cortisol secretion: Overlap is common. Eur. J. Endocrinol..

[36] Pedersini P., Turroni S., Villafañe J.H. (2020). Gut microbiota and physical activity: Is there an evidence-based link?. Sci. Total. Environ..

[37] Lou Y., Ren L., Chen H., Zhang T., Pan Q. (2024). Unveiling the hidden impact: Subclinical hypercortisolism and its subtle influence on bone health. Aging Med..

[38] Sandru F., Carsote M., Dumitrascu M.C., Albu S.E., Valea A. (2020). Glucocorticoids and Trabecular Bone Score. J. Med. Life.

[39] Chiriac O., Capitanu B.S., Gherghe M.E., Maier C., Preda E.M., Cergan R., Scheau C. (2025). Personalized Rehabilitation Following Total Knee Arthroplasty: Integrating Clinical and Imaging Perspectives. Balneo PRM Res. J..

[40] Porr C., Harris D.M., Vidrighin A., Catana A., Diaconu C., Preda E.M., Popa M.L., Berghea E.C. (2025). Etoricoxib-Induced Fixed Erythema. J. Clin. Med..

[41] Iwamoto Y., Kimura T., Morimoto Y., Sugisaki T., Dan K., Iwamoto H., Sanada J., Fushimi Y., Shimoda M., Fujii T. (2025). Development of a prediction model by combining tumor diameter and clinical parameters of adrenal incidentaloma. Endocr. J..

[42] Li X., Lan H., Lin X., Huang H., Wen J., Chen G., Lin W. (2025). Metabolic complications and clinical outcomes of non-functioning adrenal incidentalomas: A systematic review and meta-analysis. BMC Endocr. Disord..

[43] Kastelan D., Kraljevic I., Dusek T., Knezevic N., Solak M., Gardijan B., Kralik M., Poljicanin T., Skoric-Polovina T., Kastelan Z. (2015). The clinical course of patients with adrenal incidentaloma: Is it time to reconsider the current recommendations?. Eur. J. Endocrinol..

[44] Bolat Erdogan H., Gül K., Sahutoglu T., Erdogan V. (2025). Oxidative stress, antioxidant capacity, and cardiovascular risk in patients with non-functioning adrenal incidentalomas. Endocr. Connect..

[45] Morelli V., Palmieri S. (2019). Adrenal incidentaloma: Differential diagnosis and management strategies. Minerva Endocrinol..

[46] Sahdev A. (2017). Recommendations for the management of adrenal incidentalomas: What is pertinent for radiologists?. Br. J. Radiol..

[47] Morelli V., Scillitani A., Arosio M., Chiodini I. (2017). Follow-up of patients with adrenal incidentaloma, in accordance with the European society of endocrinology guidelines: Could we be safe?. J. Endocrinol. Investig..

[48] Watari J., Vekaria S., Lin Y., Patel M., Kim H., Kang F., Lubitz S., Beninato T., Laird A.M. (2022). Radiology report language positively influences adrenal incidentaloma guideline adherence. Am. J. Surg..

[49] Eldeiry L.S., Alfisher M.M., Callahan C.F., Hanna N.N., Garber J.R. (2018). The impact of an adrenal incidentaloma algorithm on the evaluation of adrenal nodules. J. Clin. Transl. Endocrinol..

[50] Nistor C., Ranetti A.E., Ciuche A., Pantile D., Constantin L.M., Brincoveanu R. (2014). Betadine in chemical pleurodesis. Farmacia.

[51] Hanna F.W.F., Issa B.G., Sim J., Keevil B., Fryer A.A. (2018). Management of incidental adrenal tumours. Br. Med. J..

[52] Espiard S., Benomar K., Loyer C., Vahé C., Vantyghem M.C. (2018). European recommendations for the management of adrenal incidentalomas: A debate on patients follow-up. Ann. Endocrinol.

[53] Lee J.-M., Kim M.K., Ko S.-H., Koh J.-M., Kim B.-Y., Kim S.W., Kim S.-K., Kim H.J., Ryu O.-H., Park J. (2017). Clinical Guidelines for the Management of Adrenal Incidentaloma. Endocrinol Metab..

[54] Inukai T., Harai N., Nakagawa Y., Hosokawa T., Antoku A., Muroi Y., Ogiwara M., Tsuchiya K. (2024). Subclinical Cushing’s Disease with High-Molecular-Weight Forms of Adrenocorticotropic Hormone Production. Case Rep. Endocrinol..

[55] Nistor C., Ciuche A., Constantinescu I. (2017). Emergency surgical tracheal decompression in a huge retrosternal goiter. Acta Endocrinol..

[56] Ren L.P., Chen H., Zhang T., Pan Q. (2023). The effect of subclinical hypercortisolism on bone metabolism. Zhonghua Nei Ke Za Zhi.

[57] Dumitrascu T., Preda E., Ionescu M. (2015). Emphysematous cystitis: An unreported complication after pancreaticoduodenectomy. Med. Surg. J. Rev. Med. Chir..

[58] Sconfienza E., Tetti M., Forestiero V., Veglio F., Mulatero P., Monticone S. (2023). Prevalence of Functioning Adrenal Incidentalomas: A Systematic Review and Meta-analysis. J. Clin. Endocrinol. Metab..

[59] Pizzorno L., Pizzorno J. (2022). Subclinical Hypercortisolism: An Important, Unrecognized Dysfunction. Integr. Med..

[60] Amado A., Torre A., Graça S.A.R., Tavares A.B. (2022). Subclinical Cushing’s syndrome: Resection of adrenal incidentaloma. BMJ Case Rep..

[61] Miller B.S., Auchus R.J. (2020). Evaluation and Treatment of Patients with Hypercortisolism: A Review. JAMA Surg..

[62] Ivović M., Marina L.V., Šojat A.S., Tančić-Gajić M., Arizanović Z., Kendereški A., Vujović S. (2020). Approach to the Patient with Subclinical Cushing’s Syndrome. Curr. Pharm. Des..

[63] Petramala L., Olmati F., Concistrè A., Russo R., Mezzadri M., Soldini M., De Vincentis G., Iannucci G., De Toma G., Letizia C. (2020). Cardiovascular and metabolic risk factors in patients with subclinical Cushing. Endocrine.

[64] Nistor C.E., Pantile D., Gavan C.S., Ciuche A. (2022). Pneumothorax on COVID-19 patients -retrospective clinical observations. Rom. J. Leg. Med..

[65] Yilmaz N., Avsar E., Tazegul G., Sari R., Altunbas H., Balci M.K. (2021). Clinical Characteristics and Follow-Up Results of Adrenal Incidentaloma. Exp. Clin. Endocrinol. Diabetes.

[66] Kanis J.A., Cooper C., Rizzoli R., Reginster J.Y. (2019). Scientific Advisory Board of the European Society for Clinical and Economic Aspects of Osteoporosis and Osteoarthritis (ESCEO); Committees of Scientific Advisors and National Societies of the International Osteoporosis Foundation (IOF). Executive summary of European guidance for the diagnosis and management of osteoporosis in postmenopausal women. Aging Clin. Exp. Res..

[67] https://www.fraxplus.org/.

[68] https://www.fraxplus.org/frax-plus.

[69] Liu M.S., Lou Y., Chen H., Wang Y.J., Zhang Z.W., Li P., Zhu D.L. (2022). Performance of DHEAS as a Screening Test for Autonomous Cortisol Secretion in Adrenal Incidentalomas: A Prospective Study. J. Clin. Endocrinol. Metab..

[70] Eller-Vainicher C., Morelli V., Aresta C., Salcuni A.S., Falchetti A., Carnevale V., Persani L., Scillitani A., Chiodini I. (2020). Defining Nonfunctioning Adrenal Adenomas on the Basis of the Occurrence of Hypocortisolism after Adrenalectomy. J. Endocr. Soc..

[71] Ahn S.H., Kim J.H., Cho Y.Y., Suh S., Kim B.J., Hong S., Lee S.H., Koh J.M., Song K.H. (2019). The effects of cortisol and adrenal androgen on bone mass in Asians with and without subclinical hypercortisolism. Osteoporos. Int..

[72] Kim B.J., Kwak M.K., Ahn S.H., Kim J.S., Lee S.H., Koh J.M. (2018). The association of cortisol and adrenal androgen with trabecular bone score in patients with adrenal incidentaloma with and without autonomous cortisol secretion. Osteoporos. Int..

[73] Cozadd A.J., Schroder L.K., Switzer J.A. (2021). Fracture Risk Assessment: An Update. J. Bone Jt. Surg. Am..

[74] Elamin Ahmed H., Al-Dadah O. (2022). Bone mineral density in fracture neck of femur patients: What’s the significance?. World J. Orthop..

[75] Sebro R., Ashok S.S. (2021). A Statistical Approach Regarding the Diagnosis of Osteoporosis and Osteopenia From DXA: Are We Underdiagnosing Osteoporosis?. JBMR Plus.

[76] Manea M.M., Dragos D., Ghenu M.I., Enache I.I., Stoican I.C., Ciulavu C., Vasiliu O., Sirbu C.A., Tuta S. (2025). The Neurocardiogenic Impact of Ischemic Stroke: Intricacies of Cardiac Enzymes and the Vegetative System. Rom. J. Mil. Med..

[77] Zavatta G., Di Dalmazi G. (2024). Mild Autonomous Cortisol Secretion (MACS)-Related Osteoporosis. Exp. Clin. Endocrinol. Diabetes.

[78] Jiménez Cassinello J.M., Vega-Beyhart A., Bernarda Iriarte M., Donato S., Herrera-Martínez A.D., Marazuela M., Araujo-Castro M. (2025). Mild autonomous cortisol secretion: Impact on bone health and quality of life. A review. Endocrine.

[79] Pal R., Banerjee M., Prasad T.N., Walia R., Bhadada T., Singh J., Bhadada S.K. (2024). Fracture risk and bone health in adrenal adenomas with mild autonomous cortisol secretion/subclinical hypercortisolism: A systematic review, meta-analysis and meta-regression. J. Bone. Miner. Res..

[80] Favero V., Eller-Vainicher C., Morelli V., Cairoli E., Salcuni A.S., Scillitani A., Corbetta S., Casa S.D., Muscogiuri G., Persani L. (2024). Increased Risk of Vertebral Fractures in Patients with Mild Autonomous Cortisol Secretion. J. Clin. Endocrinol. Metab..

[81] Nakao H., Yokomoto-Umakoshi M., Nakatani K., Umakoshi H., Ogata M., Fukumoto T., Kaneko H., Iwahashi N., Fujita M., Ogasawara T. (2023). Adrenal steroid metabolites and bone status in patients with adrenal incidentalomas and hypercortisolism. EBioMedicine.

[82] Alkan S., Guney S.C., Akcura C., Ozdemir N., Hekimsoy Z. (2025). Should adrenal incidentaloma patients be evaluated for muscle mass, function, and quality? A cross-sectional study. Endocrine.

[83] Nistor C.E., Pantile D., Stanciu-Gavan C., Ciuche A., Moldovan H. (2022). Diagnostic and Therapeutic Characteristics in Patients with Pneumotorax Associated with COVID-19 versus Non-COVID-19 Pneumotorax. Medicina.

[84] Prinzi A., Lombardo A.M., Finocchiaro S., Galvano A., Vella V., Frasca F., Malandrino P. (2025). Expanding the Clinical Profile of Mild Autonomous Cortisol Secretion: New Diagnostic Markers and Emerging Complications. Endocr. Pr..

[85] Turan Erdogan B., Evranos Ogmen B., Sacikara M., Aydin C., Topaloglu O., Ersoy R., Cakir B. (2025). The relationship between mild autonomous cortisol secretion and metabolic diseases in cases with adrenal incidentaloma. Endokrynol. Pol..

